# Mycobacteriophage SWU1 gp39 can potentiate multiple antibiotics against Mycobacterium via altering the cell wall permeability

**DOI:** 10.1038/srep28701

**Published:** 2016-06-28

**Authors:** Qiming Li, Mingliang Zhou, Xiangyu Fan, Jianlong Yan, Weimin Li, Jianping Xie

**Affiliations:** 1 Institute of Modern Biopharmaceuticals, State Key Laboratory Breeding Base of Eco-Environment and Bio-Resource of the Three Gorges Area, Key Laboratory of Eco-environments in Three Gorges Reservoir Region, Ministry of Education, School of Life Sciences, Southwest University, Beibei, Chongqing 400715, China; 2 School of Biological Science and Technology, University of Jinan, Shandong 250022, China; 3 National Tuberculosis Clinical Lab of China, Beijing Key laboratory on Drug-resistant Tuberculosis Research, Beijing Tuberculosis and Thoracic Tumor Research Institute, Beijing Chest Hospital, Capital Medical University, Beijing 101149, China

## Abstract

*M. tuberculosis* is intrinsically tolerant to many antibiotics largely due to the imperviousness of its unusual mycolic acid-containing cell wall to most antimicrobials. The emergence and increasingly widespread of multidrug-resistant tuberculosis (MDR-TB) and extensively drug-resistant tuberculosis (XDR-TB) revitalized keen interest in phage-inspired therapy. SWU1gp39 is a novel gene from mycobacteriophage SWU1 with unknown function. SWU1gp39 expressed in *M. smegmatis* conferred the host cell increased susceptibility to multiple antibiotics, including isoniazid, erythromycin, norfloxacin, ampicillin, ciprofloxacin, ofloxacin, rifampicin and vancomycin, and multiple environment stresses such as H_2_O_2_, heat shock, low pH and SDS. By using EtBr/Nile red uptake assays, WT-pAL-gp39 strain showed higher cell wall permeability than control strain WT-pAL. Moreover, the WT-pAL-gp39 strain produced more reactive oxygen species and reduced NAD^+^/NADH ratio. RNA-Seq transcriptomes of the WT-pAL-gp39 and WT-pAL revealed that the transcription of 867 genes was differentially regulated, including genes associated with lipid metabolism. Taken together, our results implicated that SWU1gp39, a novel gene from mycobacteriophage, disrupted the lipid metabolism of host and increased cell wall permeability, ultimately potentiated the efficacy of multiple antibiotics and stresses against mycobacteria.

Tuberculosis, caused by *Mycobacterium tuberculosis* (*M. tuberculosis*), remains a major global public health concern. The emergence of multidrug-resistant *M. tuberculosis* (MDR-MTB) and extensively drug-resistant *M. tuberculosis* (XDR-MTB) strains exacerbated the situation. *M. tuberculosis* is intrinsically tolerant to many antibiotics largely due to the imperviousness of its unusual mycolic acid-containing cell wall to most chemotherapeutics and inducible expression of several active drug efflux pumps. Some genes involved in metabolism or other processes were identified to mediate intrinsic resistance in mycobacteria. The inactivation of asparagine synthetase AsnB, an asparagine biosynthetic enzyme catalyzing the transfer of the γ -amino residue of glutamine to the carboxyl residue of aspartate, dramatically sensitized *Mycobacterium smegmatis* (*M. smegmatis*) to multiple antibiotics, including rifampin, erythromycin, novobiocin and fusidic acid^1^. Mycobacteria proteasome accessory facror (*pafC*), a component of bacterial proteasome, was identified to be involved in fluoroquinolones intrinsic resistance. PafC inactivation mutants are specifically hypersensitive to fluoroquinolones, including moxifloxacin, norfloxacin, ofloxacin, ciprofloxacin, but not to other antibiotics such as isoniazid, rifampicin, spectinomycin, chloramphenicol, capreomycin^2^. Protein kinase G (pknG), Serine/threonine protein kinase, was also required for intrinsic antibiotic resistance in mycobacteria^3^. In *M. smegmatis*, another Serine/threonine protein kinase *MSMEG_5437* and a predicated anti-sigma factor *MSMEG_6129* were involved in intrinsic resistance to a variety of stresses including the genotoxic agent mitomycin C, hydrogen peroxide and at least four different antibiotics, namely isoniazid, chloramphenicol, erythromycin and tetracycline^4^. Isocitrate lyases (ICLs), metabolic enzymes previously recognized as dedicated to the replenishing of tricarboxylic acid (TCA) cycle intermediates, was recently shown to have unexpected role in antioxidant defense as a mechanism of antibiotic tolerance^5^. Cytochrome *bd* was found to play a key role in the survival of mycobacteria during multiple lethal stress, including hydrogen peroxide, clofazimine and bedaquiline^6^,^7^. The intrinsic drug resistance of mycobacteria represents formidable obstacle to tuberculosis control. It is imperative to find novel therapeutic or synergetic targets that can potentiate the antimicrobial lethality against tuberculosis.

Despite the highly impermeable mycolic acid containing cell wall, several first-line or second-line anti-tuberculosis drugs targeting cell wall are in clinical use, such as Isoniazid (INH) which can disrupt the cell wall integrity by inhibiting mycolic acid biosynthesis^8^. Upon entry into *M.tuberculosis*, the prodrug INH is activated to form an isonicotinyl-free radical by the bacterial peroxidase/catalase katG^9^. The INH-free radical reacts with NAD to form an INH-NAD complex that is a highly potent inhibitor of the essential enzyme InhA, an enoyl-ACP reductase required for mycolic acid biosynthesis^10^,^11^. Multiple factors contributing to clinical isolates INH resistance limited the efficacy of INH^12^,^13^. Ethionamide, a structural analog of INH, is another anti-TB drug that inhibits InhA activity by a mechanism of action similar to that of INH but requires activation by EthA instead of katG^10^. Mutations in the inhA structural gene or promoter region underpin the resistance to ETH. Other factors such as alterations in the drugs activators expression, redox status change, drug inactivation, and drug efflux pump activation^14^, contribute to the resistance to INH and ETH. New anti-TB drugs targeting cell wall are needed to combat tuberculosis.

Bacteriophage, the virus of bacteria, is a useful tool to control bacteria infections. Endolysins are peptidoglycan hydrolases encoded by bacteriophages that can break down bacterial peptidoglycan at the terminal stage of the phage reproduction cycle^15^. Endolysins are in trial as potential antimicrobial agents, especially for multidrug-resistant bacteria. Abp1, a virulent phage targeting the multidrug-resistant *Acinetobacter baumannii*, encoding an endolysin PlyAB1 exhibited significant antibacterial activity against all 48 clinical PDRAB isolates within a relatively short time frame^16^. Many mycobacteriophage-derived endolysins have shown antimycobacterial activity, including mycobacteriophage Bxz2^17^, BTCU-1^18^, Ms6^19^,^20^, D29^21^. The lytic endolysins are intensively pursued as alternative sanitation or disinfectant agents against mycobacterium infections. With an estimated huge biomass of 10^31^ phages worldwide, phage-encoded endolysins and other effective proteins represent vast reservoir for novel antimicrobials. Bacteriophage P1-encoded Ref protein, a novel class of endonuclease, is toxic specifically during the bacterial SOS response or the stationary phase cultures^22^. These studies implicate that phage-based therapy can be alternative or complement to antibiotics.

In this study, we identified a novel gene gp39 from mycobacteriophage SWU1, which was absent in mycobacteriophage L5, although SWU1 and L5 are highly similar. We expressed SWU1gp39 in *M. smegmatis* using vector *pALACE* under the control of an acetamide inducible promoter. The recombinant strain (WT-pAL-gp39, induced by acetamide) showed growth deficiency in medium containing hygromycin in comparison with the control strain (WT-pAL), although *pALACE* contains a hygromycin resistance cassette. SWU1gp39 expressed in *M. smegmatis* leads to increased susceptibility to multiple antibiotics, including isoniazid, erythromycin, norfloxacin, ampicillin, ciprofloxacin, ofloxacin, rifampicin and vancomycin, also some environmental stresses such as H_2_O_2_, SDS, low pH and heat shock. Using RNA-seq, we found that the regulatory and metabolic pathways were changed by SWU1gp39. Scanning electron microscopy (SEM) and ethidium bromide or nile red uptake assays showed the morphology and cell wall permeability alteration in the recombinant strain.

## Results

### Gp39 is a novel gene from Mycobacteriophage SWU1

**Figure 1:**
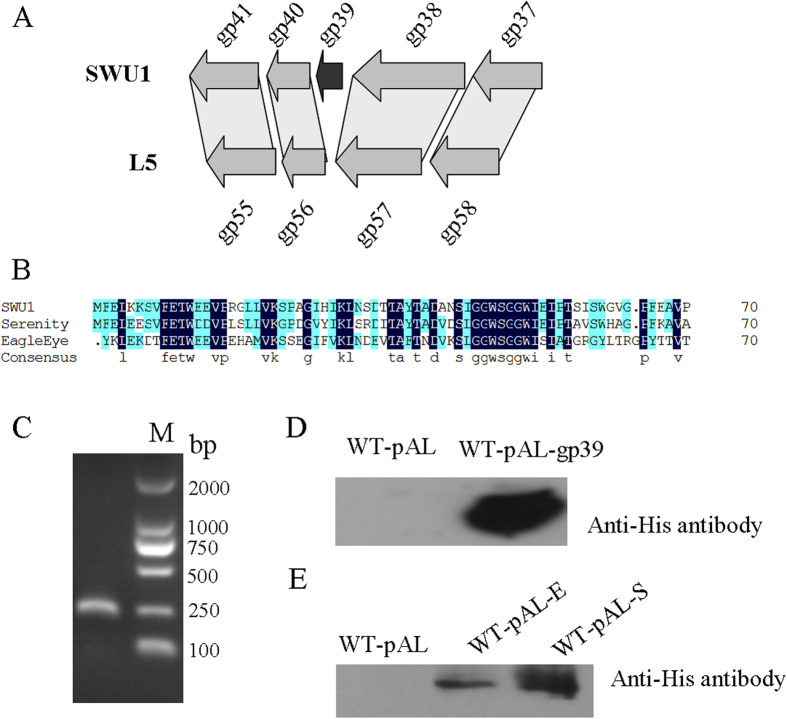
The expression of a novel gene from mycobacteriophage SWU1 in *M. smegmatis*. (**A**) Comparison of the organization of the gp39 genome locus in SWU1 and related species L5. The grey shading represents region of conservation between genomes. (**B**) Amino acid sequence alignment (generated using DNAMAN) of gp39 in SWU1 and close relative. (**C**) PCR amplification of gp39 encoding sequence approximately 240 bp. The SWU1gp39 gene (**D**) and its homologous genes from Serenity or EagleEye (**E**) were expressed in *M. smegmatis* and detected using Western blotting.

Mycobacteriophage SWU1 is a newly isolated phage from soil sample collected in Sichuan province, China^23^, which is highly similar to mycobacteriophage L5^24^, but no homolog of SWU1gp39 was found in the L5 genome (Fig. 1A). No homolog was found in NCBI using BLAST until mycobacteriophage EagleEye and Serenity were isolated (Fig. 1B). To define the function of SWU1gp39, it was amplified from SWU1 genome (Fig. 1C) and expressed a His-tagged gp39 protein using a recombinant pALACE plasmid (Fig. 1D).

### SWU1gp39 expression affects host growth upon exposure to several antibiotics

**Figure 2:**
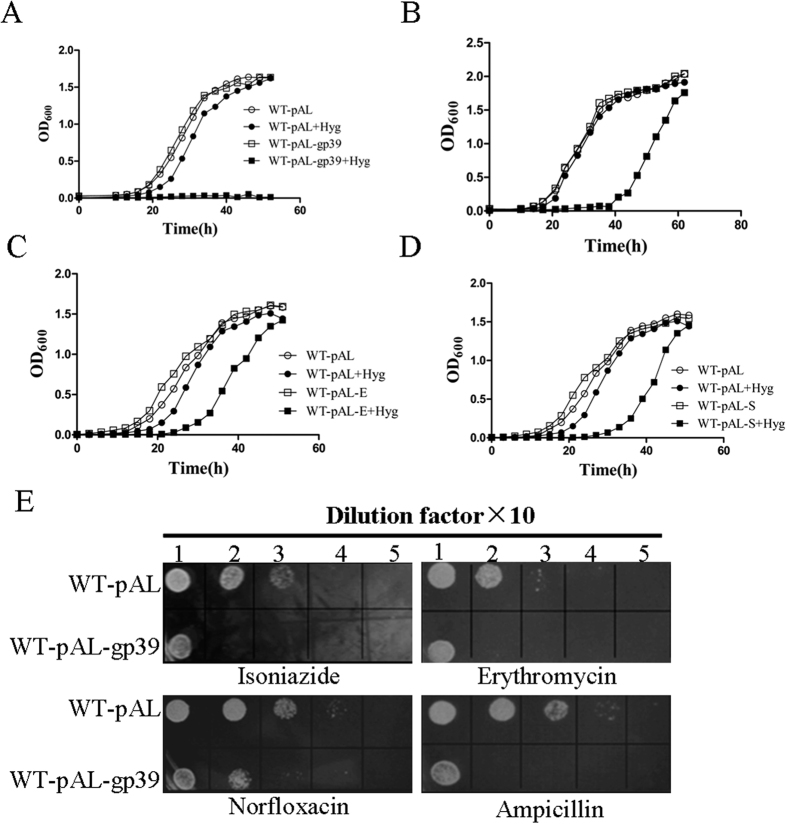
WT-pAL-gp39 increased bacterial growth defect following exposure to multiple antibiotics. The bacterial strains of WT-pAL, WT-pAL-gp39 (**A**), WT-pAL-E (**C**) and WT-pAL-S (**D**) were grown in M9 medium (**B**) or Middlebrook 7H9 medium supplemented with 0.05% Tween 80, 0.2% glycerinum and 0.25% acetamide, with or without hygromycin (100 μg/ml). The OD_600_ were determined at an interval of 3 h. (**E**) Ten-fold serial dilutions of WT-pAL and WT-pAL-gp39 were spotted on Middlebrook 7H10 containing isoniazide (4 μg/ml), erythromycin (16μg/ml), norfloxacin (1μg/ml) and ampicilin (100 μg/ml). Then the result was recorded when incubated at 37 °C for 3 days. The data reported represent the means (n = 3) ± SD (standard deviation).

To test the effect of SWU1gp39 on recombinant strains, the growth rates of WT-pAL and WT-pAL-gp39 were monitored at OD_600_ after initial inoculation at the same concentration. As shown in Fig. 2, no growth differences were detected between WT-pAL and WT-pAL-gp39, demonstrating that the expression of SWU1gp39 does not affect the growth of *M. smegmatis* in 7H9 medium supplemented with 0.05% Tween 80 and 0.2% glycerinum. In the presence of Hyg, WT-pAL cells started to grow and entered into logarithmic phase 3h delay in comparison to the absence of Hyg. In contrast, WT-pAL-gp39 treated with Hyg was incapable of growth though pALACE containing a Hyg resistance cassette (Fig. 2A). Similar growth defect was found when WT-pAL and WT-pAL-gp39 grown in M9 medium supplemented with glucose as the only carbon source, however WT-pAL-gp39 strain can grow to logarithmic phase after 60 h (Fig. 2B). To determine whether the homologous genes from EagleEye and Serenity possess similar function, the related genes were synthesized by commercial company and expressed in *M. smegmatis* by using the same shuttle plasmid pALACE (named WT-pAL-E and WT- pAL-S) (Fig. 1E). As expected, WT-pAL-E and WT- pAL-S also showed some growth defect in the presence of Hyg (Fig. 2C,D). The growth rates were also tested when WT-pAL and WT-pAL-gp39 exposed to other antibiotics on the plates. As shown in Fig. 2E, prominent growth defect was observed upon exposure to isoniazid, erythromycin, norfloxacin and ampicillin. Though *M. smegmatis* possess intrinsic resistance to amplicillin due to its cell wall complex structure, overexpressed SWU1gp39 can potentiate the antibiotic sensitivity.

### Expression of SWU1gp39 in *M. smegmatis* can potentiate multiple anti-tuberculosis drugs

**Figure 3:**
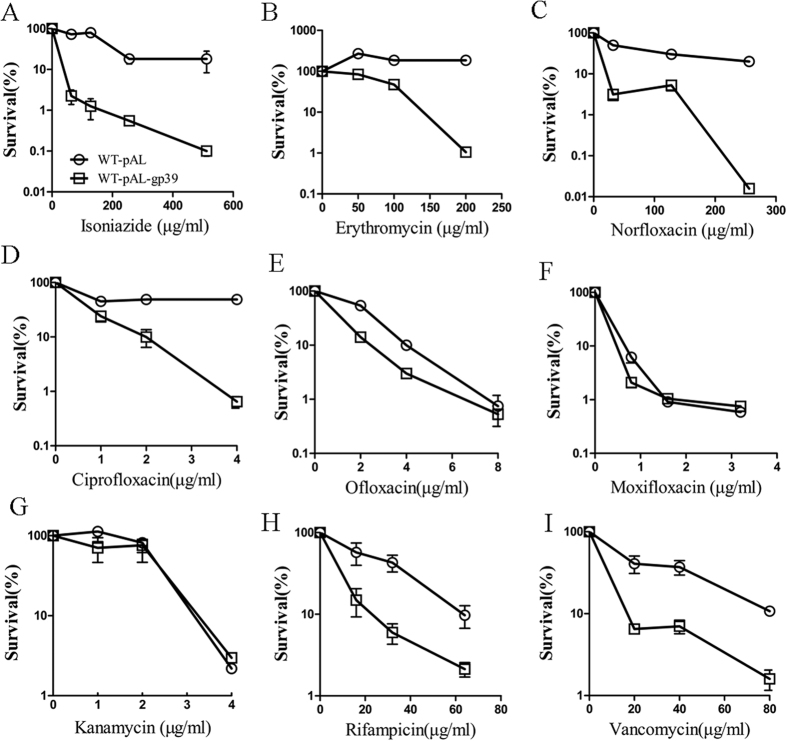
SWU1gp39 expression reduced bacterial survival following exposure to lethal stress. The control strain (WT-pAL, OD_600_ = 1) and recombinant strain (WT-pAL-gp39, OD_600_ = 1) were diluted in 7H9 medium and then treated with isoniazid for 12 h (**A**), erythromycin for 24 h (**B**), norfloxacin for 4 h (**C**), ciprofloxacin for 4 h (**D**), ofloxacin for 4 h (**E**), moxifloxacin for 2 h (**F**), kanamycin for 2 h (**G**), rifampicin for 12 h (**H**) or vancomycin for 10 h (**I**) as the indicated concentrations. Symbols: empty circle, WT-pAL; empty squares, WT-pAL-gp39. Experiments were performed three times and similar results were obtained, error bars indicate standard deviation.

WT-pAL-gp39 has growth defect in the presence of multiple antibiotics, including isoniazid, erythromycin, norfloxacin and ampicillin. To determine whether expression of SWU1gp39 will compromise the survival during lethal antibiotics stress, we exposed WT-pAL and WT-pAL-gp39 cells to various concentrations of several antimicrobial compounds (Fig. 3). First, we analysed the effect of isoniazid, the survival of WT-pAL-gp39 was 10–100 folds lower than that of WT-pAL (Fig. 3A). Similar phenomenon was observed when WT-pAL and WT-pAL-gp39 were treated with various concentrations of erythromycin for 24 h and norfloxacin for 4 h (Fig. 3B,C). These data indicate that SWU1gp39 protein contributes to the lethal activity of isoniazid, erythromycin and norfloxacin against wild type cells. To better understand the role of SWU1gp39 in bacteria upon harsh stress, another three fluoroquinolones were tested on WT-pAL and WT-pAL-gp39. Differential survival rates were found when WT-pAL and WT-pAL-gp39 treated with ciprofloxacin (Fig. 3D) and ofloxacin (Fig. 3E), however no difference was found when treated with moxifloxacin (Fig. 3F). Kanamycin, an aminoglycoside antibiotic, kill bacteria dependent on ROS, but no differences were found when WT-pAL and WT-pAL-gp39 were treated with kanamycin. To discriminate whether SWU1gp39 affects cell wall or membrane stress in mycobacteria, vancomycin, another antibiotic blocking cell wall synthesis, was tested. Vancomycin binds to the terminal D-ala-D-ala of the pentapeptide chain on the peptidoglycan precursor molecules on the outside of the cell membrane, thus affecting only the cell wall synthesis machinery in bacteria. Killing curve experiments revealed WT-pAL-gp39 are more susceptibility towards vancomycin than WT-pAL, indicating that SWU1gp39 most likely affects the cell wall integrity.

To determine the sensitivity of WT-pAL-gp39, MICs of WT-pAL and WT-pAL-gp39 were determined in 7H9 liquid medium at 37 °C using the broth dilution method containing 0.25% acetamide. The results confirmed that WT-pAL-gp39 is sensitive to isoniazid, norfloxacin, ciprofloxacin, ofloxacin, nalidixic acid, erythromycin (Table 1). MICs of WT-pAL-gp39 are between 2- and 8-fold lower than WT-pAL for these antibiotics (Table 1). Sensitivity of WT-pAL-gp39 to other antibiotics, including moxifloxacin, kanamycin, capreomycin, remained unchanged.

### Gp39 expression leads to increased *M. smegmatis* sensitivity to various environmental stresses

**Figure 4:**
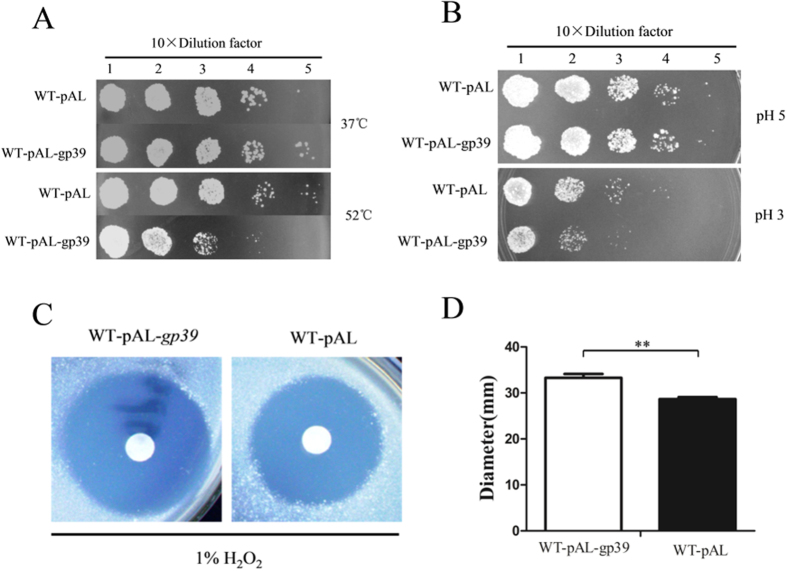
Gp39 expression leads to increased *M. smegmatis* sensitivity to various environmental stresses. (**A**) Survival of WT-pAL and WT-pAL-gp39 after treatment with heat shock. (**B**) Survival of WT-pAL and WT-pAL-gp39 after treatment with low pH. (**C**) Disc diffusion assay was performed using discs containing 1% H_2_O_2_. (**D**) The diameter zone of complete inhibition was measured. The data reported represent the means (n = 3) ± SD.

Environment stress factors are important for mycobacterium growth, the success of *M. tuberculosis* within host necessitates handling of various environment stresses, such as low pH, oxidative stresses. To determine whether gp39 expression also affects the bacterial response to common environmental stresses, we compared the response of WT-pAL-gp39 strain and empty vector control strain to various stresses. The WT-pAL-gp39 strain showed reduced survival following exposure to 52 °C for 20 min (Fig. 4A) and low pH for 6 h (Fig. 4B) in contrast to the empty vector control. To determine the effect of hydrogen peroxide on WT-pAL-gp39 and WT-pAL, disk diffusion was used (Fig. 4C). The WT-pAL-gp39 strain was more sensitive to redox stress via hydrogen peroxide treatment relative to WT-pAL, as the zone of inhibition was significantly larger for the SWU1gp39 expression when compared to vector control (Fig. 4D). Taken together, these results showed that SWU1gp39 expression increased *M. smegmatis* sensitivity to various environment stresses.

### Gp39 expression regulates the transcription of multiple *M. smegmatis* genes

**Figure 5:**
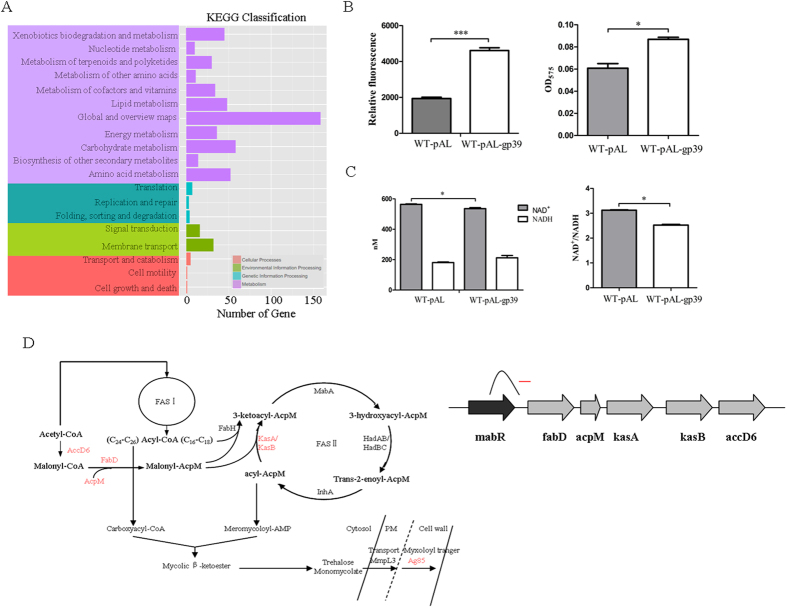
(**A**) KEGG classification analysis of transcriptional data of WT-pAL and WT-pAL-gp39. (**B**) ROS determined in WT-pAL and WT-pAL-gp39. The data reported represent the means (n = 3) ± SD. (**C**) Comparison of NAD^+^ and NADH levels in WT-pAL and WT- pAL-*gp39*. The data reported represent the means (n = 3) ± SD. (**D**) The FAS I and FAS II pathways in mycobacteria.

In order to gain insight into the regulatory changes in *M. smegmatis* expressed SWU1 gp39, we compared the global transcription alteration of the recombinant strain (WT-pAL-gp39) and the empty vector (WT-pAL) control strain during mid-log phase by using RNA-Seq transcriptome sequencing. A total of 867 genes (details are listed in the Supplementary materials) showed significant differential transcription in WT-pAL-gp39, including 511 downregulated genes and 356 upregulated genes. Based on gene ontology and KEGG analysis, the recombinant strain showed differential expression of a wide array of genes involved in metabolic process (Fig. 5). SWU1gp39 expressed in *M. smegmatis* potentiated multiple anti-tuberculosis drugs lethality, but few genes were found to be directly involved in antibiotics except *MSMEG_6440* (*Rv3854c*, ethA), *MSMEG_5312*, *MSMEG_1420* and *MSMEG_5102* (Table 2). ETH is a prodrug, activated by the NADPH-specific flavin ademine dinucleotide-containing monooxygenase EthA (*MSMEG_6440* or *Rv3854c*). We found that WT-pAL-gp39 showed some growth defect on the solid medium containing indicated concentration of ETH (Fig. S1). This might be due to upregulated EthA expression or more activated ETH. To our knowledge, these genes can not explain why WT-pAL-gp39 became more sensitive to multiple antibiotics. Interestingly, the WT-pAL-gp39 significantly downregulated genes involved in lipid metabolism, including fadD, acpM, kasA, kasB, accD6 (Table 2). Mycolic acids are major and specific long-chain fatty acids essential for the *M. tuberculosis* cell envelope. The biosynthesis of mycolic acid precursors requires two types of fatty acid synthesis (FASs), the eukaryotic-like multifunctional enzyme FAS I and the acyl carrier protein (ACP)-dependent FAS II systems (Fig. 5D). The genes encoding the FAS II enzymes are distributed in three independent transcription units^25^. The first is formed by fabD-acpM-kasA-kasB-accD6 (Fig. 5D), and all of them are downregulated in WT-pAL-gp39. MabR (mycolic acid biosynthesis regulator) was originally identified as a putative transcriptional regulator encoded by *MSMEG_4324* (*Rv2242*) and located immediately upstream of the main *Fas* II operon. Overexpression of MabR in *M. smegmatis* represses the transcription of *fad*D, *acp*M, *kas*A, *kas*B and was accompanied by reduced levels of mycolic acids^26^. Several genes involved in lipid degradation were also upregulated in WT-pAL-gp39 (Table 2). These results suggested that SWU1gp39 might affect the biosynthesis of cell wall mycolic acids components.

### Gp39 expression induces redox-related physiological alterations

Our previous transcriptome of SWU1gp39 recombinant strain was associated with altered global metabolism. Antibiotics lethality was reported to be related with redox-related physiological alterations^27^. In order to characterize the redox potential of SWU1gp39 recombinant strain and vector, DCFH-DA was used to monitor the ROS level. DCFH-DA readily penetrates bacterial cells^28^,^29^; once entry the cells, DCFH-DA is converted by cellular esterases into a nonmembrane-permeating compound that can be oxidized to a fluorescent form by ROS^28^. Bacterial cell were treated with 10 μM DCFH-DA and then fluorescence intensity were detected with excitation at 488 nm and emission at 515 nm. As shown in Fig. 5B, the recombinant strain exhibited higher fluorescence labeling than the vector control strain. The ROS levels between WT-pAL-gp39 and WT-pAL were also confirmed by second method described below.

As FAS II utilize NADH as cofactors and multiple NAD+ metabolism related genes were downregulated in recombinant strain based on transcriptomic data (see TABLE S1 in the supplemental material), expression of SWU1gp39 in *M. smegmatis* might change the cellular NAD+/NADH level. Therefore, the cellular NAD+ and NADH level were measured. The results showed that SWU1gp39 expression decreased the cellular NAD+ level in WT-pAL-gp39 cells in comparison to WT-pAL (Fig. 5C). Meanwhile, the level of NADH in WT-pAL-gp39 cells was slightly higher than that in WT-pAL. The ratio of NAD+/NADH was 20% lower in WT-pAL-gp39 than that in WT-pAL (Fig. 5C).

### Gp39 expression affects surface motility and biofilm formation

**Figure 6:**
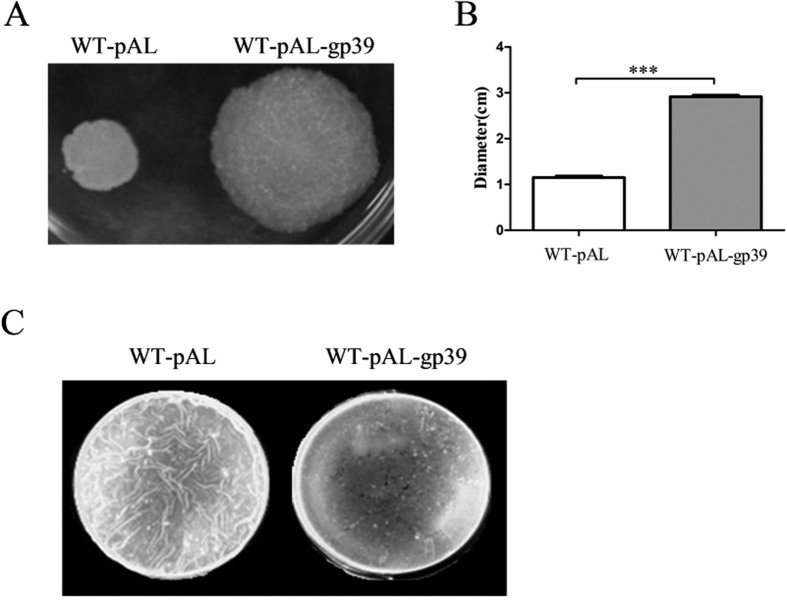
Effect of SWU1gp39 on surface motility and biofilm growth. (**A**) Sliding motility of *M. smegmatis* on M63 medium. (**B**) The diameter zome of the motility colony. The data reported represent the means (n = 3) ± SD. (**C**) Surface biofilm growth of *M. smegmatis* strain.

**Figure 7:**
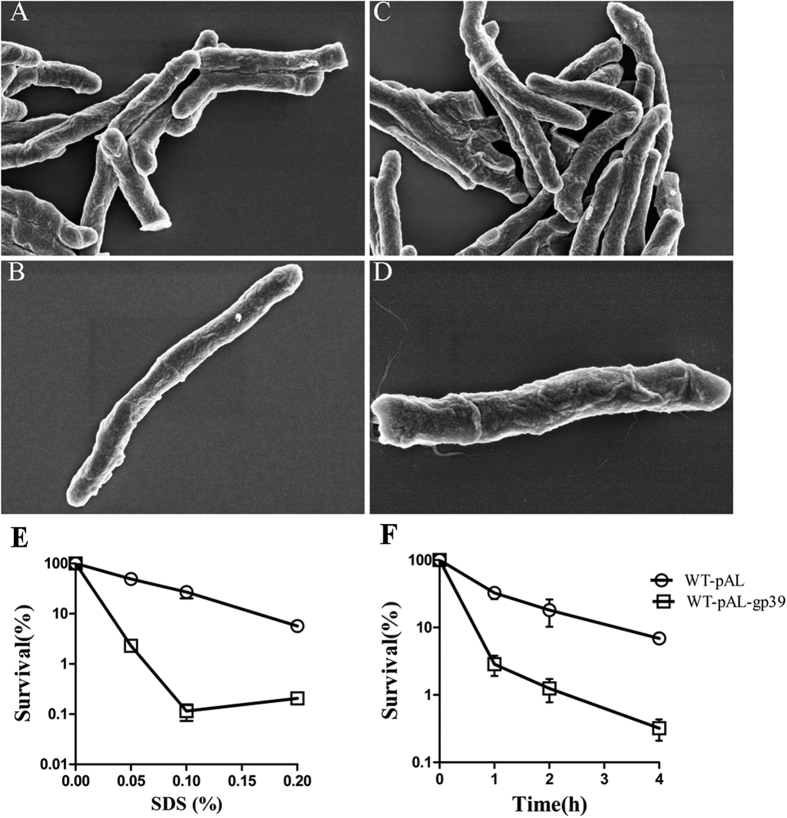
Scanning electron micrographs of WT-pAL (**A**,**B**) and WT-pAL-gp39 (**C**,**D**). The effects of SWU1gp39 on bacterial survival after treatment with SDS as indicated concentration (**E**) and time (**F**). Symbols: empty circle, WT-pAL; empty squares, WT-pAL-gp39. Experiments were performed three times and similar results were obtained, error bars indicate standard deviation.

Because the SWU1gp39 expression showed susceptibility to multiple antibiotics targeting cell wall, we determined whether gp39 can affect the sliding motility and biofilm formation. Surprisingly, WT-pAL-gp39 exhibit enhanced sliding on the surface of minimum medium agar plates in comparison with WT-pAL (Fig. 6A,B). In some pathogenic bacteria, such as *Vibrio cholerae*, *Pseudomonas aeruginosa*, *Haemophilus influenza*, *Streptococcus* sp., *Escherichia coli* and *M. tuberculosis*, host persistence and antibiotic tolerance are closely correlated with the ability to form biofilms^30^. At day 3 after inoculation, the culture of control strain has already colonized the whole surface of the liquid medium, whereas the recombinant strain only formed sporadic clumps in the liquid phase and a few islands of growth on the surface (Fig. 6C). These results are reminiscent of an effect of SWU1gp39 on the cell wall of mycobacterium. Scanning electron microscopy did not show strikingly difference between WT-pAL-gp39 and WT-pAL, but showed more pits in the surface of WT-pAL-gp39 (Fig. 7A–D and Fig. S2). Consistent with a defective cell wall, WT-pAL-gp39 became more sensitive to the detergent sodium dodecyl sulphate (SDS) upon treatment with different time and concentration (Fig. 7E,F). WT-pAL-gp39 was at least 10–100 folds more susceptible to SDS than WT-pAL.

### The cell wall permeability of *M. smegmatis* Gp39 recombiant is altered

*M. smegmatis* with SWU1gp39 expression was more sensitive to various hydrophobic antibiotics, including erythromycin, norfloxacin, ciprofloxacin, ofloxacin, and rifampicin, as well as isoniazid and vancomycin which are hydrophilic (Fig. 3). However, the sensitivity to other hydrophobic or hydrophilic antibiotics, moxifloxacin, kanamycin, streptomycin, capreomycin and chloramphenicol, remained unchanged (Table 1).

**Figure 8:**
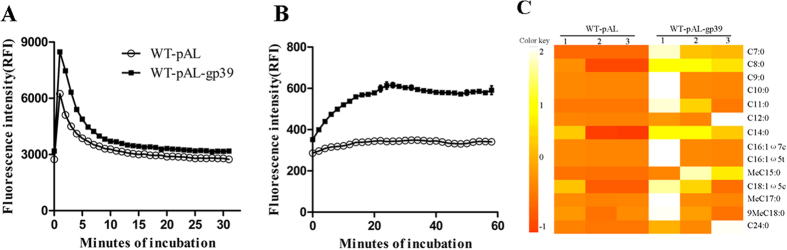
SWU1gp39 expression in *M. smegmatis* results in increased cell wall permeability. (**A**) Mid-log-phase cultures of WT-pAL (OD_600_ = 0.5) control and WT- pAL-gp39 (OD_600_ = 0.5) strains were incubated in PBS with 25 mM glucose and 2μg/ml ethidium bromide. The data reported represent the means (n = 3) ± SD. (**B**) Mid-log-phase cultures of WT-pAL control and WT-pAL-gp39 strains were incubated in PBS containing 25 mM glucose and 2μM Nile red stain. The data reported represent the means (n = 3) ± SD. (**C**) Fatty acid extracted from WT-pAL and WT-pAL-gp39 analyzed by gas chromatography mass spectrometry (GC-MS). Symbols: empty circle, WT-pAL; filled squares, WT-pAL-gp39.

To examine whether the increased drug sensitivity of the abovementioned recombinant bacteria is caused by a general increase in cell permeability, we used fluorescence spectroscopy to measure the whole-well accumulations of ethidium bromide and Nile Red, representatives of the hydrophilic and hydrophobic compounds, respectively^31^. The results showed that both compounds accumulated more rapidly and to higher levels in the recombinant strain than the empty vector control strain, indicating an increase in cell wall permeability (Fig. 8A,B).

Mycolic acids are major and specific long-chain fatty acids essential for mycobacterium dell envelope. Mycobacteria possess two fatty acid synthase systems, the eukaryotic-like fatty acid synthase typeI (FAS I) and the prokaryotic FAS II. Mycobacterial FAS I synthesizes fatty acids in a bimodal pattern, which are elongated by the FAS II system to produce mycolic acids, long-chain fatty acids, which are the major constituents of the mycobacterial cell wall. FAS I and FAS II utilize NADPH and NADH as cofactors. Our data showed that multiple genes encoding the FAS II enzymes were downregulated and NADH cofactor accumulated, suggesting that FAS II might be affected by SWU1gp39. To explore whether the expression of SWU1gp39 in *M. smegmatis* can change the composition of fatty acids, gas chromatography mass spectrometry (GC-MS) was used to detect the fatty acids of WT-pAL-gp39 and WT-pAL. There are 14 major compounds identified in WT-pAL-gp39 and WT-pAL, from C7 to C24 (Fig. 8C). Several compounds were found accumulated in WT-pAL-gp39, especially C7:0, C8:0, C11:0, C14:0, MeC15:0, C18:1ω5(c), C24:0 (Fig. 8C). The data indicated that SWU1gp39 can alter the relative abundance of fatty acids in *M. smegmatis*.

## Discussion

Multidrug-resistant M. tuberculosis (MDR-MTB) and extensively drug-resistant M. tuberculosis (XDR-MTB) represent formidable challenge to global public health. New effective drug or small-molecule potentiators of bacteriocidal antibiotics are urgently needed^32^. Peptidoglycan recognition proteins (PGRPs) are a family of evolutionary conserved antibacterial innate immunity proteins^33^. PGRPs kill antibiotic-resistant bacteria, synergistic targeting of oxidative, thiol, and metal stress can be used for the development of new approaches to kill antibiotic resistant bacteria^34^. Vitamin C (VC) is an essential nutrient for some mammals and potent antioxidant. The activity of VC against drug-susceptible, MDR- and extensively drug-resistant (XDR) *M. tuberculosis* was associated with the presence of high iron concentration, reactive oxygen species (ROS) production and DNA damage. Phage, as a nemesis of the bacteria, is a useful tool for scientific research. Phages were highly expected for the treatment of drug-resistant bacteria.

In this study, we showed that SWU1gp39 expressed *M. smegmatis* showed change of sensitivity to multiple antibiotics, including isoniazid (INH), erythromycin (ERY), fluoroquinolones, rifampicin (RIF), vancomycin (VAN), ampicillin (AMP) when. Isoniazid is a pro-drug, targeting enoyl ACP reductase InhA after activated by the catalase-peroxidase KatG^10^. Erythromycin is a macrolide antibiotic targeting the 50S ribosome and inhibiting bacterial protein synthesis^35^. Fluoroquinolones is a class of synthetic drugs, which target the gyrase (gyrA or gyrB) and also function via the secondary effect of lethal doses of hydroxyl radicals produced^36^. Rifampicin is first-line anti-tuberculosis drug, inhibiting the transcription of *M. tuberculosis* by binding to the beta subunit of the RNA polymerase (RpoB) encoded by the *rpoB* gene^37^,^38^. Rifampicin also induced hydroxyl radical formation^39^. Vancomycin and ampicillin inhibit bacteria growth by targeting bacteria cell wall and also lead to the production of hydroxyl radical^40^. Our transcriptome data showed that the transcription of most genes associated with antibiotics action did not changed by gp39 (Table 2), suggesting a negligible role of WT-pAL-gp39 in regulating the transcription of genes known to be associated with antibiotics action. WT-pAL-gp39 showed growth defect in the presence of hygromycin in comparison to WT-pAL even though pALACE contains a hygromycin resistance cassette. We speculated that WT-pAL-gp39 possess higher cell wall permeability in comparison with WT-pAL.

The intracellular survival of *M. tuberculosis* entails extraordinary capability to tackle diverse stresses. Our data showed that WT-pAL-gp39 can weaken the Mycobacteria ability to handle multiple environment stresses including low pH, heat shock and hydrogen peroxide, which are relevant to antibiotics efficacy^41^,^42^. Cell wall defect can be found in some mutants, such as acid-sensitive mutants^43^, exopolyphosphatase Rv1026/PPX2^44^ and lipoprotein mutation^45^. The survival ability of WT-pAL-gp39 was 10- to 100- fold reduced in comparison to that of WT-pAL cells when exposed to SDS for various times or various concentration, suggesting cell wall defect in WT-pAL-gp39. Multiple antibiotics targeting cell wall were discovered, it is tempting to explore whether these cell wall targeting antibiotics have similar sensitizing effect. Congo red, a dye binding to the lipoproteins present on the mycobacterial surface^46^, was used to characterize modifications affecting the cell wall^45^. After 4 days of growth on a solid medium containing Congo Red at 37 °C (without Tween80), no difference was observed between the two *M. smegmatis* strains (Fig. S3). Biofilm plays crucial role in antibiotic tolerance and persistence in *M. tuberculosis*^47^. In this study, we found that SWU1gp39 expression was associated with reduced biofilm formation, but increased in surface motility. This is consistent with previous results that WT-pAL-gp39 antibiotic susceptibility is associated with the environment stressor and biofilm, but not Congo Red uptake.

This work showed for the first time that mycobacteriophage SWU1gp39 protein altered the cell wall permeability, manifested by increased uptake of the polar compound EtBr and the lipophilic dye Nile red. The intrinsic resistance of mycobacteria to most antimicrobial agents is generally attributed to an unusual cell wall dominated by lipids and carbohydrates, which provides a barrier to noxious compounds and limits drug uptake^48^,^49^. Previous studies have shown that mycobacterial uptake of Nile red is directly related to cell wall lipid components and susceptibility to lipophilic drugs^50^,^51^. Mycobacterium cell wall permeability for EtBr also is closely related with antibiotic resistance^52^. We conclude that WT-pAL-gp39 can sensitize the recombinant to multiple stresses via increasing the cell wall permeability. More accumulation of short-chain fatty acids in WT-pAL-gp39 than in the WT-pAL suggested that SWU1gp39 disrupted normal synthesis of long-chain fatty acids, the major components of mycobacteria cell wall.

Phage therapy was renewed as promising alternative to antibiotics for tackling resistant pathogens^53^. Engineered bacteriophage with protein targeting gene networks overexpressed can boost the bactericidal effect of antibiotics^54^. For therapeutic applications of phage, SWU1gp39 might be used as a broad-spectrum antibiotic adjuvant or potentiator, in addition to be included in engineered bacteriophage to enhance bacterial killing by antibiotics, especially first-line anti-tuberculosis drugs such as isoniazed and rifampicin.

## Methods

### Bacteria strain, plasmid, and growth conditions

*M. smegmatis* mc^2^155 and recombinant bacteria grown in Middlebrook 7H9 medium supplemented with 0.05% Tween80 and 0.2% glycerinum or Middlebrook 7H10 plates supplemented with 0.5% glycerinum. Luria-Bertani medium was used to culture *E. coli* strains. Antibiotics were added at following concentrations: ampicillin, 100 μg/ml; kanamycin, 50 μg/ml for *E. coli* and 20 μg/ml for *M. smegmatis*; hygromycin, 75 μg/ml for *E. coli* or 50 μg/ml for *M. smegmatis*. All cultures were incubated at 37 °C.

### Expression of His-tagged SWU1gp39 protein

The open reading frame encoding *SWU1 gp39* gene was amplified by PCR from *SWU1* genomic DNA using the forward primer 5′ ATGGATCCATGTTCGAACTG3′ containing a *Bam*HI site (underlined) and the reverse 5′ AATCGATTCACAGGGGCACCGCTT3′ containing a *Cla*I site (underlined). The PCR product of approximately 250 bp was cloned into *Mycobacteria*-*Escherichia coli* shuttle plasmid *pALACE*. The sequence verity was confirmed by DNA sequencing.

### Bacterial growth curves

Recombinant strains WT-pAL and WT-pAL-*gp39* were cultured in 7H9 medium containing 0.5% glycerol, 0.05% Tween 80 and 0.25% acetamide until OD_600_ reached 0.8–1.0. Then the cultures were reinoculated in fresh 7H9 medium at the ratio of 1:1000 dilution. Cultures were incubated at 37 °C with shaking through the entire growth phase. Samples were collected at the same growth stage, and the OD_600_ values were measured every 3 h after growth initiation. Experiments were performed in triplicates, and the average values were used to generate growth curves.

### Survival curves

Mid-exponential phase cultures of WT-pAL and WT-pAL-*gp39* were diluted in 7H9 medium and grown at 37 °C treated for various concentrations as indicated with antibiotics. After indicated time, surviving cells were estimated by colony formation on drug-free agar. The percentage cfu recovered was determined relative to an untreated control sampled at the time when antibiotics added. All the experiment was repeated at least three times.

### Disk diffusion method

The disk diffusion method was used to qualitatively measure the differences in H_2_O_2_ or SDS sensitivities between WT-pAL and WT-pAL-*gp39* mycobacterium. Mid-exponential-phase cultures were used to prepare the lawns of cells as previously described^55^. An indicated concentration of H_2_O_2_ or SDS was spotted on 5.5 mm-diameter Whatman filter disks placed on the bacterial lawn. After overnight incubation at 37 °C, the diameter of zone of complete inhibition was measured. All the experiment was repeated at least three times.

### Spot tests

WT-pAL and WT-pAL-*gp39* were grown to an OD_600_ of 0.8–1.0 tested for their susceptibility to antibiotics by spotting a 10-fold serial dilution initially on Middlebrook 7H10 containing 0.25% acetamide and a range of drugs. The concentration of antibiotics for spot tests as following described: isoniazid (4 μg/ml), erythromycin (16 μg/ml), norfloxacin (1 μg/ml), ampicillin (100 μg/ml).

### MIC determination

The MIC of anti-TB drugs were determined as previously described^2^. Briefly, broth dilution with visual inspection of a series of tubes each containing about 10^5^ bacteria in 1 ml of 7H9 medium supplemented with concentrations of drug increasing by 2 times increments and 0.25% acetamide. Following 3 days incubation at 37 °C, the lowest concentration that prevented visible growth was defined as the MIC.

### Heat shock and acid challenge

Mid-exponential phase cultures of WT-pAL and WT-pAL-*gp39* washed with phosphate buffer (pH 7.0), and then diluted OD_600_ = 0.5. For heat shock, the bacteria were incubated in 52 °C for 20 min, then serially diluted in phosphate buffer and spotted onto 7H9 plates. For acid challenge studies, the bacteria were incubated in low pH medium for 6 h, then serially diluted in phosphate buffer and spotted onto 7H9 plates.

### RNA-seq

Following exposure of logarithmically growing cultures (WT-pAL and WT-pAL-*gp39*, OD_600_ = 0.8) to the inducer acetamide for at least 4 h, RNA was harvested using Trizol-based methods. RNA samples were treated with DNase and purified RNA was got by using RNA clean kit (TIANGEN). The samples were sent to Shanghai Biotechnology Corporation for library construction and sequencing using Illumina Hi seq 2500 (Illumina). The sequence quality of the data sets was checked by Agilent 2100 Bioanalyzer. The transcriptome sequencing (RNA-seq) data were aligned with the *M. smegmatis* mc^2^155 genome obtained from NCBI (http://www.ncbi.nlm.nih.gov/genome/?term = MC2_155). Gene expression level was estimated by the reads number. In order to make it comparable between different genes or samples, reads number were converted into FPKM (Fragments Per Kilobase of exon model per Million mapped reads) for the normalization of gene expression^56^. Every gene Fragments number was calculated after TopHat alignment by HTseq^57^, Then normalization was carried out using TMM (trimmed mean of M values)^58^ and the FPKM was calculated by Perl script.

### Gene ontology and KEGG classification analysis

Gene Ontology (GO) annotation transcriptome was performed using the UniProt-GOA Database (http://www.ebi.ac.uk/GOA/). Proteins were categorized into biological process, cellular compartment and molecular function according to Gene Ontology annotation. Kyoto Encyclopedia of Genes and Genomes (KEGG) were utilized to annotate pathways: firstly, using KEGG online service tools KAAS to annotate proteins, secondly, using KEGG online service tools KEGG mapper to map on the KEGG pathway database, finally, using InterPro database and InterProScan to annotate protein domains and applying CORUM database to annotate protein complex.

### Scanning electron microscopy study

WT-pAL and WT-pAL-gp39 cells were grown for about 24 h until the OD_600_ reaches 1.0 in 7H9. Cultures were harvested by centrifugation and the harvested pellets were then resuspended in 2.5% glutaraldehyde solution. The samples were dehydrated in an ascending series of ethanol. After critical point drying, samples were sputtered with platinum and observed by a scanning electron microscopy (SEM; FEI Quanta 200).

### Ethidium bromide accumulation/efflux assay and Nile red uptake assays

Strains of mycobacterium were grown in 7H9 to an OD_600_ = 1.0, washed twice with phosphate-buffered saline (PBS, 137 mM NaCl, 4.3 mM Na_2_HPO_4_, 1.4 mM KH_2_PO_4_, pH 7.0) and resuspended. The resuspended cells were determined and adjusted to 0.4 and containing 25 mM glucose. After standing 5 minutes, 200 μl of the suspension cells was added in triplicate to a 96-well black fluoroplate and Nile Red and EtBr were added to final concentrations. The accumulation of these dyes was measured with an excitation of 540 nm and emission of 630 nm for Nile Red and an excitation of 545 nm and emission of 600 nm for EtBr.

### ROS determination in planktonic cells

The intracellular production of ROS was detected by the reduction of nitro blue tetrazolium (NBT) (Sigma) to nitro blue diformazan. Bacterial suspensions (500 μl of stationary phase) were inoculated with 500 μl of NBT (1mg/ml) at 37 °C for 30 min. Bacterial cells were separated from the supernatant by centrifugation at 1500 g for 10 min, and then treated with 600 μl DMSO and 800 μl of PBS, pH 7.0. Reduced NBT was measured as formazan blue at 575 nm.

The second method we used for ROS determination was Reactive Oxygen Species Assay Kit (Beyotime Institute of Biotechnology, China) based on 2′,7′-dichlorodihydrofluorescein deacetate (DCFH-DA) to determine the generation of ROS. We acquired cell samples in logarithmic phase and stained them with 10 μM DCFH-DA. We estimated the fluorescence intensity with excitation at 488 nm and emission at 515 nm.

### NAD^+^ and NADH concentration assay

NAD^+^ and NADH concentrations were measured using a NAD^+^/NADH Assay kit (BioAssay Systems). M. smegmatis culture was diluted to OD_600_ of 0.5 and then washed twice with cold phosphate buffer saline (PBS). Cell pellets were resuspended with 100 μl of NAD^+^ extraction buffer for NAD^+^ measurement and 100 μl of NADH extraction buffer for NADH measurement. Then extracts were heated at 60 °C for 5 min. Supernatant was collected for the EnzyChrom NAD^+^/NADH Assay kit.

### Fatty acid analysis

The Mid-exponential phase cultures *M. smegmatis* fatty acid were extracted as previously described^59^. The concentration of fatty acid was determined by an Agilent 7890A gas chromatograph with 5975C mass selective detector (GC-MSD) equipped with an Agilent 7693A automatic liquid sampler and a DB-5MS capillary column. The detailed analysis procedure was described previously^60^.

### Statistical analysis

Data from at least three biological replicates were used to calculate means and standard deviation (SD) for graphing purposes. Statistical analysis employed the unpaired student’s *t* test, asterisks indicate statistically significant difference (*P < 0.05; **P < 0.01; ***P < 0.001).

## Additional Information

**How to cite this article**: Li, Q. *et al*. Mycobacteriophage SWU1 gp39 can potentiate multiple antibiotics against Mycobacterium via altering the cell wall permeability. *Sci. Rep.* **6**, 28701; doi: 10.1038/srep28701 (2016).

## Supplementary Material

Supplementary Information

Supplementary Information

## Figures and Tables

**Table 1:** MIC of various antibiotics for WT-pAL and WT-pAL-*gp39.*

Strain	MIC(μg/ml)													
	INH	NOR	CIP	MOX	OFL	NAL	ERY	KAN	RIF	VAN	AMP	STR	CAP	CHL
WT-pAL	8	8–16	1	0.2	0.5	256	100	0.25	4	2.5	1600	0.5	1.25	32
WT-pAL-gp39	4	2	0.25	0.2	0.25	128	25	0.25	4	1.25	800	0.5	1.25	32

INH, isoniazid; NOR, norfloxacin; CIP, Ciprofloxacin; MOX, Moxifloxacin; OFL, Ofloxacin; NAL, Nalidixic acid; ERY, Erythromycin; KAN, Kanamycin; RIF, Rifampicin; VAN, Vancomycin; AMP, Ampicillin; STR, Streptomycin; CAP, Capreomycin; CHL, Chloramphenicol.

**Table 2:** Genes differentially regulated in WT-pAL-*gp39* compared with WT-pAL.

Category	Gene ID	Fold change	Homologs	Gene description
Drug resistance related genes	MSMEG_6440	16.9	Rv3854c	monooxygenase, flavin-binding family protein
	MSMEG_5312	2.59		multidrug ABC transporter ATPase
	MSMEG_1420	2.42		transcriptional regulatory protein
	MSMEG_5102	3.22		ABC transporter ATP-binding protein
lipid metabolism	MSMEG_4324	7.45	Rv2242	MabR, mycolic acid biosynthesis regulator
	MSMEG_4327	−4.82	Rv2245	KasA, involved in meromycolate extension
	MSMEG_4326	−4.86	Rv2244	AcpM, lipid metabolism
	MSMEG_4328	−4.14	Rv2246	KasB, lipid metabolism
	MSMEG_4325	−3.75	Rv2243	FadD, lipid metabolism
	MSMEG_4329	−3.50	Rv2247	AccD6, lipid metabolism
	MSMEG_3580	−2.70	Rv0129c	lipid metabolism
	MSMEG_6179	−2.12	Rv3667	lipid metabolism
	MSMEG_2078	−2.01	Rv1886c	lipid metabolism
	MSMEG_1387	3.64	Rv0672	involved in lipid degradation
	MSMEG_1388	4.16	Rv0673	enoyl-CoA hydratase EchA4
	MSMEG_1390	3.39	Rv0675	enoyl-CoA hydratase EchA5
	MSMEG_5996	3.24	Rv3546	involved in lipid degradation
	MSMEG_4717	3.00	Rv2502c	fatty acid metabolism
	MSMEG_6041	2.41	Rv3573c	lipid degradation
	MSMEG_3465	2.18	Rv1925	lipid degradation
	MSMEG_4716	2.51	Rv2501c	lipid metabolism
	MSMEG_5907	2.15	Rv3505	involved in lipid degradation
	MSMEG_5923	2.10	Rv3523	lipid carrier protein
	MSMEG_5922	2.18	Rv3522	lipid transfer protein
