# Supplementary material for: Mycobacteriophage SWU1 gp39 can potentiate multiple antibiotics against Mycobacterium via altering the cell wall permeability
Source: Sci Rep. 2016 Jun 28;6:28701. doi: 10.1038/srep28701 (PMC4923848; doi:10.1038/srep28701)
Supplement: Supplementary Information [file srep28701-s1.pdf]

Mycobacteriophage SWU1 gp39 can potentiate multiple antibiotics against Mycobacterium via altering the cell wall permeability

Qiming Li[1,4], Mingliang Zhou[1,4], Xiangyu Fan[1,2,4], Jianlong Yan[1], Weimin Li[3*], Jianping Xie[1*]

[1]Institute of Modern Biopharmaceuticals, State Key Laboratory Breeding Base of Eco-Environment and Bio-Resource of the Three Gorges Area, Key Laboratory of Eco-environments in Three Gorges Reservoir Region, Ministry of Education, School of Life Sciences, Southwest University, Beibei, Chongqing 400715, China. [2]School of Biological Science and Technology, University of Jinan, Shandong 250022, China. [3]National Tuberculosis Clinical Lab of China, Beijing Key laboratory on Drug-resistant Tuberculosis Research, Beijing Tuberculosis and Thoracic Tumor Research Institute, Beijing Chest Hospital, Capital Medical University, Beijing 101149, China.

[*]Address correspondence to jianping xie, georgex@swu.edu.cn; Weimin Li, lwn_18@aliyun.com

[4]These authors contributed equally to this work.

Table S1. Genes differentially regulated associated with NAD^+/ NADH in WT-pAL-gp39 compared with WT-pAL.

NAD^+/ NADH	nuoN	2.47	Rv3158	NADH dehydrogenase subunit N
	nuoG	2.16	Rv3151	NADH dehydrogenase subunit G
	nuoD	2.45	Rv3148	NADH dehydrogenase subunit D
	MSMEG_3813	2.77		NAD-dependent epimerase/dehydratase
	nuoH	2.32	Rv3152	NADH dehydrogenase subunit H
	nuoI	2.89	Rv3153	NADH dehydrogenase subunit I
	MSMEG_2059	2.89	Rv3149	NADH dehydrogenase subunit E
	nuoM	2.2	Rv3157	NADH dehydrogenase subunit M

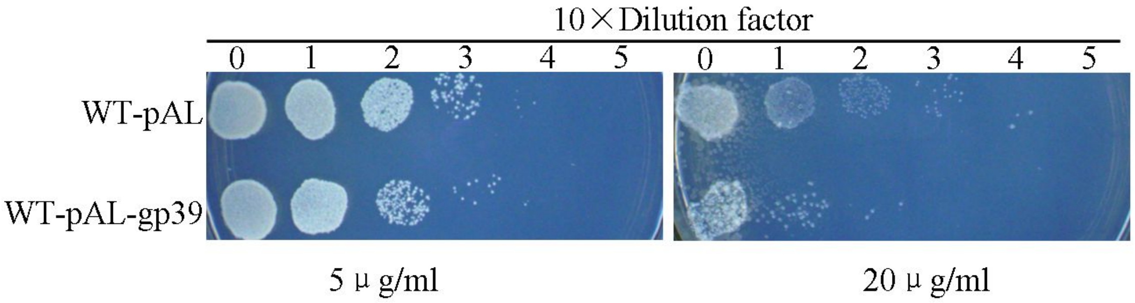

Figure S1 Ten-fold serial dilutions of WT-pAL and WT-pAL-gp39 were spotted on Middlebrook 7H10 containing ethionamide (5μg/ml and 20μg/ml).

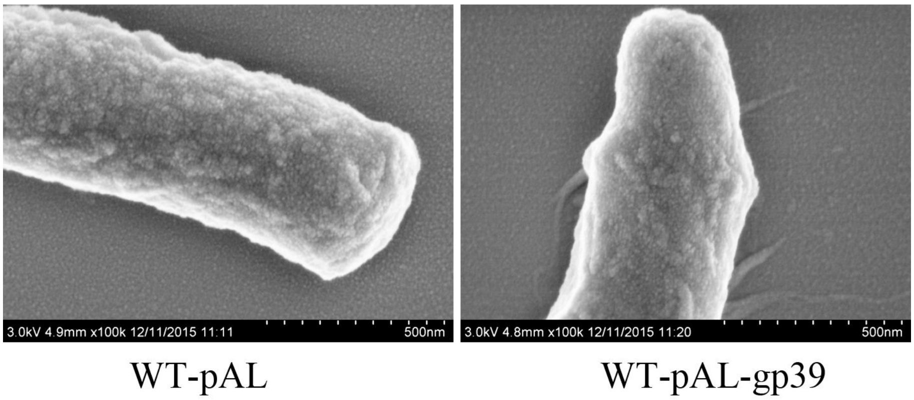

Figure S2 Scanning electron micrographs of WT-pAL and WT-pAL-gp39.

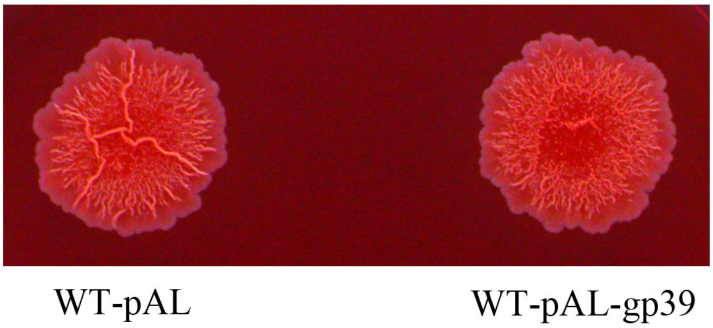

Figure S3 Congo red stain of WT-pAL and WT-pAL-gp39.
